# An Integrative Framework to Guide Social Engagement Interventions and Technology Design for Persons With Mild Cognitive Impairment

**DOI:** 10.3389/fpubh.2021.750340

**Published:** 2022-01-14

**Authors:** Elizabeth A. Lydon, Lydia T. Nguyen, Qiong Nie, Wendy A. Rogers, Raksha A. Mudar

**Affiliations:** ^1^Department of Speech and Hearing Science, University of Illinois Urbana-Champaign, Champaign, IL, United States; ^2^iN2L, Greenwood Village, CO, United States; ^3^Department of Kinesiology and Community Health, University of Illinois Urbana-Champaign, Champaign, IL, United States

**Keywords:** mild cognitive impairment, social engagement, social isolation, loneliness, technology, social activity, social network

## Abstract

Social isolation and loneliness in older adults are associated with poor health outcomes and have been linked to an increased risk of cognitive impairment and incident dementia. Social engagement has been identified as a key factor in promoting positive health behaviors and quality of life and preventing social isolation and loneliness. Studies involving cognitively healthy older adults have shown the protective effects of both in-person and technology-based social engagement. However, the benefits of social engagement for people who are already at-risk of developing dementia, namely those with mild cognitive impairment (MCI), have yet to be elucidated. We present a narrative review of the literature, summarizing the research on social engagement in MCI. First, we identified social networks (quality, size, frequency, and closeness) and social activities (frequency, format, purpose, type, and content) as two overarching dimensions of an integrative framework for social engagement derived from literature examining typical cognitive aging. We then used this framework as a lens to examine studies of social engagement in MCI to explore (i) the relationship between in-person and technology-based social engagement and cognitive, emotional, and physical health, and (ii) interventions that target social engagement including technology-based approaches. Overall, we found that persons with MCI (PwMCI) may have different levels of social engagement than those experiencing typical cognitive aging. Moreover, in-person social engagement can have a positive impact on cognitive, emotional, and physical health for PwMCI. With respect to activity and network dimensions in our framework, we found that cognitive health has been more widely examined in PwMCI relative to physical and emotional health. Very few intervention studies have targeted social engagement, but both in-person and technology-based interventions appear to have promising health and well-being outcomes. Our multidimensional framework of social engagement provides guidance for research on characterizing the protective benefits of social engagement for PwMCI and informs the development of novel interventions including technology-based approaches.

## Introduction

Social isolation is the objective state of having few social ties or infrequent social interactions and is a critical public health issue that affects nearly a quarter of adults aged 65 years and older (1). Loneliness is the subjective experience of feeling isolated or not belonging and affects ~43% of older adults (2). A growing body of evidence has linked social isolation and loneliness to significant health risks including increased mortality (3), morbidity (4, 5), and negative psychosocial outcomes (6–8). Moreover, factors linked to social isolation and loneliness in cognitively normal older adults, including small social networks, reduced participation in social activities, and poor social support, are associated with a 40–50% increased risk of developing dementia, even when physical activity, education, and depression are statistically controlled (9, 10). For populations who are already at a high risk of developing dementia, such as those with mild cognitive impairment (MCI), understanding the impact of social isolation and loneliness on disease progression and finding effective interventions is of particular importance.

MCI is an intermediate stage between normal aging and dementia, characterized by a modest decline in cognition that is greater than expected for an individual's age and education, but with relatively preserved ability to carry out daily living activities [e.g., eating, bathing; (11–13)]. MCI affects roughly 17% of people over age 60, with prevalence markedly increasing across the lifespan (14). Persons with MCI (PwMCI) are more likely to experience progressive cognitive decline compared to cognitively normal older adults, with an annual conversion rate to dementia of 10–15% (12, 15). This risk is compounded by the fact that PwMCI may experience social disengagement due to cognitive challenges making it more difficult to have fulfilling social interactions (16). Given the significant public health and economic impact of dementia with approximately 300 billion dollars spent on caring for persons with dementia in the US alone (17), addressing potentially modifiable risk factors such as social isolation and loneliness will be crucial to address this growing crisis.

Social engagement has been identified as a key target in addressing social isolation and loneliness and is defined as participation in social activities and maintenance of social connections with others (18, 19). There is a vast body of work examining the effects of increasing social engagement opportunities among cognitively normal older adults. These studies have found promising outcomes such as increased social support, higher levels of social activity, reduced feelings of loneliness, and improved psychological well-being associated with increased social engagement (20–24); for review see (25).

Additionally, there is evidence to suggest that social engagement may be protective against cognitive decline and incident dementia (18, 26–28) and may even lead to better cognitive functioning (29) and increased cognitive reserve (30, 31). The majority of these studies have examined in-person opportunities for social engagement; however, advances in technology with more affordable options becoming available have provided new ways for individuals to engage socially with others from the comfort of their own homes, and have served as a lifeline during the COVID-19 pandemic (32, 33). Studies conducted pre-pandemic showed that technologies such as videoconferencing, social networking, and social robots had the potential to increase social engagement among older adults [for reviews see (34–36)], a matter that has become increasingly important in light of the recent pandemic.

Although the cognitive aging literature generally supports the role of social engagement as a protective health factor and shows promise for social engagement interventions, the benefits of in-person and technology-based social engagement for PwMCI are not well established. Thus, the goal of this narrative review was to summarize the research on social engagement in MCI, in particular the relationship between social engagement and various health factors and the efficacy of social engagement interventions.

Social engagement is a multidimensional construct, with numerous components that could differentially relate to health. For example, one study using multiple measures of social engagement (social activity frequency, social network size, and social support) found that activity frequency and social support were more strongly associated with cognitive health than social network size, suggesting distinct mechanisms of action (29). Therefore, as part of this review, we first operationalized social engagement and its various components based on how it has been defined and measured in the literature among cognitively normal older adults. We then developed a framework of social engagement based on this body of published research to organize and guide our review. Using this framework, we examined studies of social engagement in PwMCI that fell into two broad categories: (i) studies exploring the relationships between social engagement and various health factors (cognitive, emotional, and physical); and (ii) intervention studies that have targeted social engagement.

## Guiding Framework

**Figure 1:**
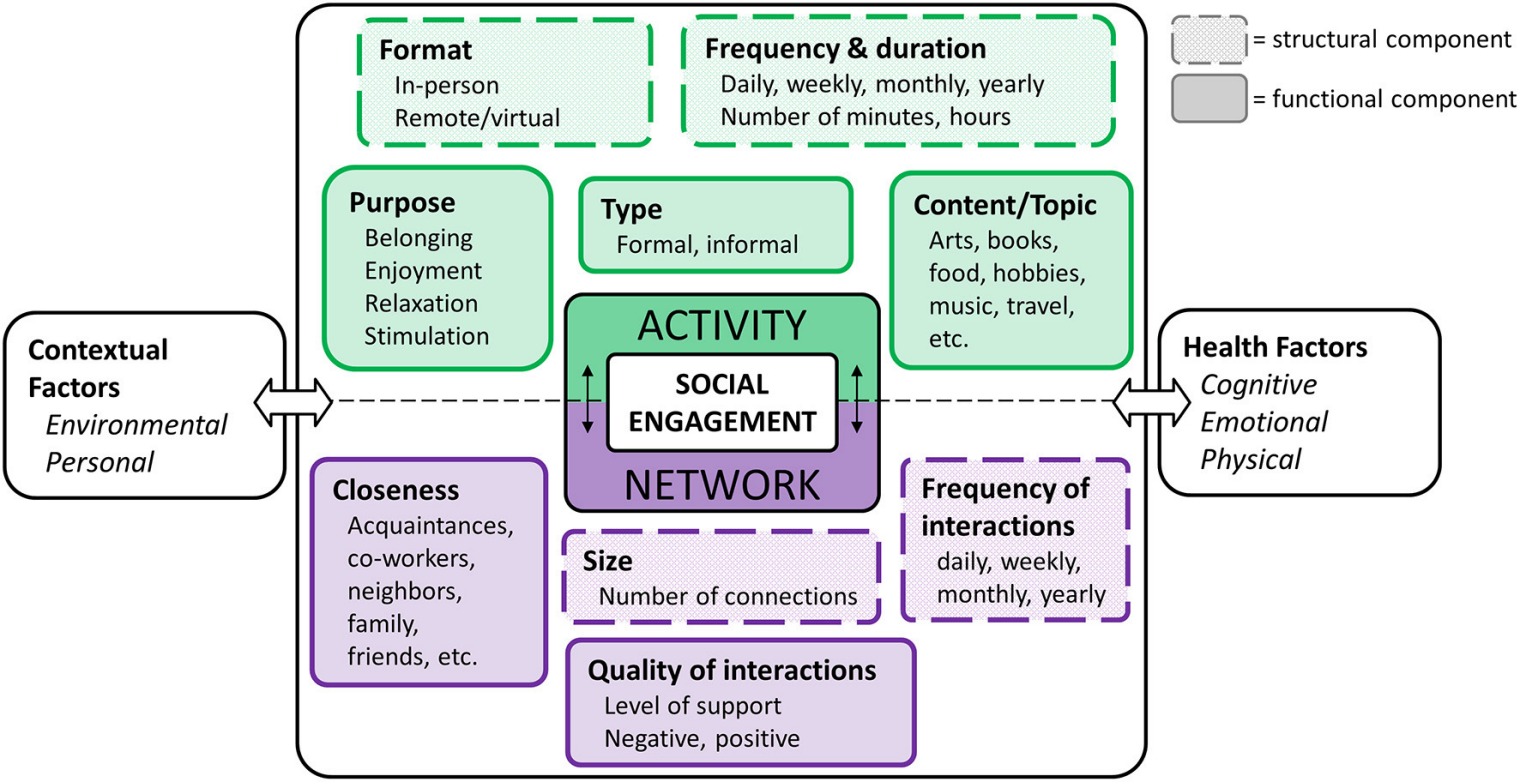
Integrative social engagement framework.

Social engagement includes two broad dimensions: participation in social *activities* and maintaining a social *network*, or social connections (18, 19). In our social engagement framework (Figure 1), we represent *social activity* and *social network* as two related dimensions, each of which can be further characterized across various *structural* and *functional* components (37). Structural components relate to form or makeup of activities and networks (e.g., frequency) and are characterized by objective measures, whereas functional components relate to what these activities and networks provide an individual (e.g., quality) and are described by qualitative characteristics (1, 3). Our framework also represents the role of health and contextual factors guided by the International Classification of Functioning, Disability, and Health [ICF; (46)]. The dimensions and components are described below.

### Activity

Social activity refers to participation in a task or event that involves some level of interaction with others and can be characterized by the structural components of frequency/duration and format, and the functional components of type, purpose, and content/topic.

The **frequency and duration** of social activity is typically measured across a range of different activities [e.g., going on a trip, going to restaurants, work, volunteering; (25)]. Such activities can occur in-person or in a virtual (i.e., technology-based) **format**. The degree to which an individual is engaged in a given activity often depends on the **type** of activity being performed. Activities that involve participation with an organized group or association can be described as formal (e.g., volunteer work, political organizations, religious groups), whereas informal activities tend to occur casually with others, for leisure [e.g., attending a concert, playing a game, visiting friends; (38–40)]. Similarly, older adults participate in activities for various reasons. For example, having a phone conversation with a friend, attending an exercise class, and volunteering at a local food bank are each likely to serve a different **purpose** and may relate to fulfillment of different social roles (41, 42). Activity **content/topic**, although not often addressed in the literature, characterizes the specific subject matter that is enjoyed or discussed during an activity.

### Network

Social network refers to the relationships, or social connections, in a person's life (43). A person's network is characterized based on structural components of **size (**i.e., how many social connections a person has) and **frequency (**i.e., how often one interacts with any given social network member). Network is also described by the functional components of **closeness**, which refers to the proximity of social network members (e.g., family member, acquaintance), and the **quality** of those interactions (e.g., positive, negative). Importantly, the structural components of a person's network are often used to define the objective state of social isolation (1), whereas the functional components contribute to one's perception of social support. Having reliable, positive interactions contribute to overall well-being, but primarily negative interactions tend to increase stress and feelings of loneliness (44).

### Contextual Factors

Contextual factors are known to impact a person's ability to remain socially engaged (45). According to the ICF, these contextual factors include both environmental and personal factors (46). **Environmental factors** typically refer to circumstances that are out of a person's control, or that occur externally to the individual. Such factors may include access to services and community (e.g., urban vs. rural), the infrastructure that exists in a given area (e.g., presence of community centers), how much social capital an individual has (e.g., economic status, inequity), and shared life events (e.g., hurricane, pandemic). On the other hand, **personal factors** typically refer to determinants that are internal to the individual, including age, race, gender, education level, coping style, etc. (46). Although not typically the focus of social engagement studies, these factors are often included as covariates in analyses.

### Health Factors

Health factors can be subdivided into three general categories: cognitive, emotional, and physical. **Cognitive health** refers to a person's ability to think, learn, and remember, and is typically measured across a range of cognitive domains, including attention, language, memory, and executive functioning (e.g., reasoning, self-control). Distinct from cognitive health, **emotional health** refers to one's experience of emotional states and feelings (both positive and negative), interest in life, and life satisfaction that supports the subjective feeling of emotional and psychological well-being. Poor emotional health may contribute to symptoms related to depression and anxiety (47, 48). Finally, **physical health** refers to the functioning of the body (internally and externally) such as mobility, sensory abilities, and vascular health.

Health factors may play a role both as an antecedent to and a consequence of social engagement, depending on the direction of the relationship. For example, a person experiencing cognitive challenges may experience a reduction in their level of social activity, where change in health is impacting the level of social engagement (49). Alternatively, those who do not participate in social activities may be at an increased risk of developing cognitive challenges, wherein health is impacted by the level of social engagement (8).

### Overview of Review

The goal of this review was to characterize the relationship between social engagement and cognitive, emotional, and physical health for PwMCI, and to assess the existing evidence from interventions that targeted social engagement in this population. Our social engagement framework (Figure 1) was developed to guide the review by allowing us to (i) logically organize the results of our review, (ii) describe the factors that have been addressed in the literature, and (iii) identify gaps in the literature to inform future research. The findings of this review can be utilized to develop appropriate in-person and technology-based social engagement interventions for PwMCI.

## Method

### Article Identification and Selection

**Figure 2:**
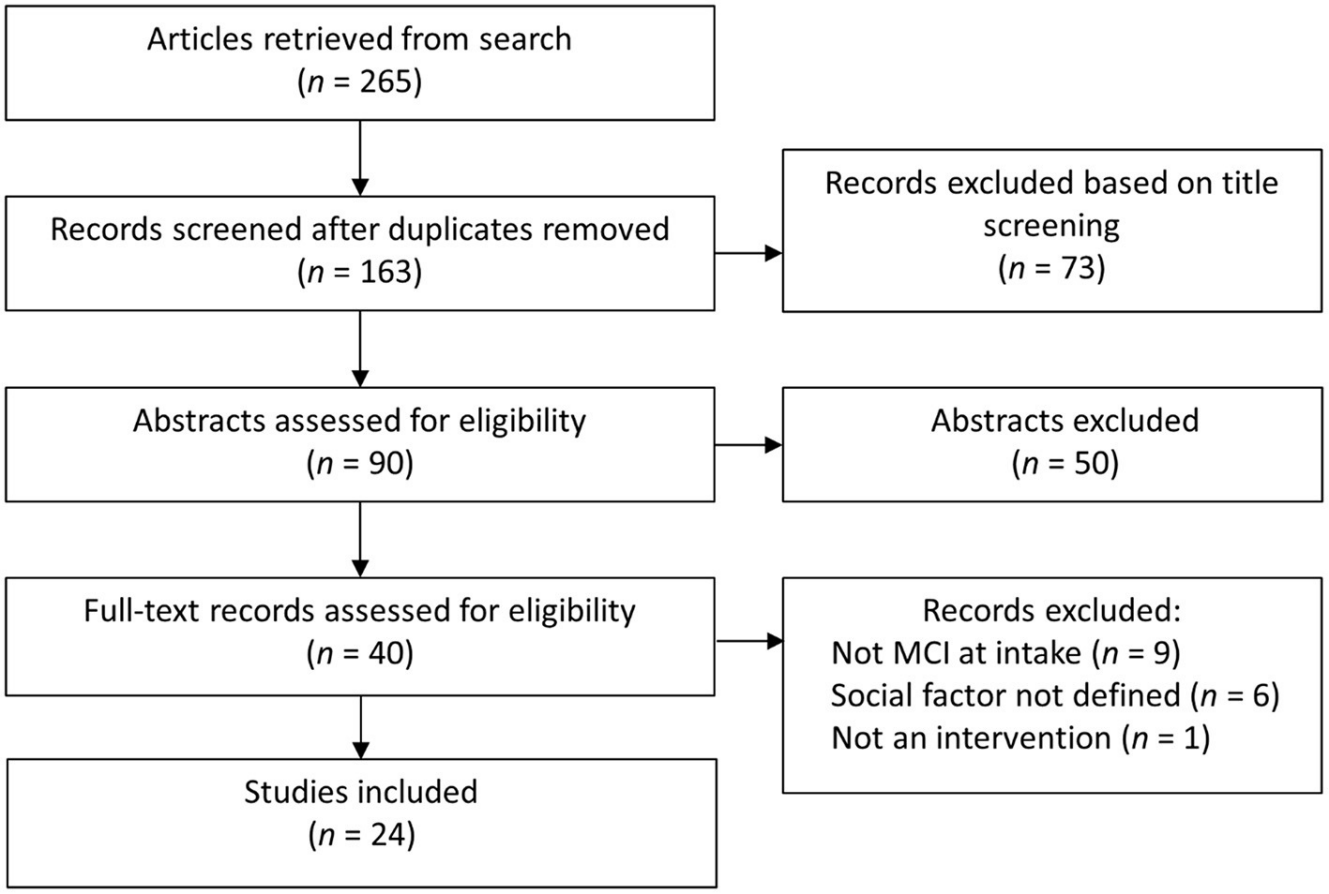
Overview of article selection.

We conducted a search of the literature using Medline and PsycINFO, including peer-reviewed journal articles published between January 2000 and July 2021. We combined the search terms “mild cognitive impairment” AND “social ___ [engagement, connections, activity, network, support].” Article titles were initially screened for duplicates and relevance (e.g., review articles; non-MCI sample). We then reviewed the remaining full-text articles and only included articles that (i) included PwMCI at intake (or well-known alternatives such as cognitive impairment no dementia), and (ii) addressed at least one factor of social engagement from our framework (Figure 1). The first two authors (EL; LN) independently reviewed all full text articles identified by the search to determine whether they met the review criteria. Any discrepancies in their decisions were discussed by all the authors to reach a consensus. The selection of articles is illustrated in Figure 2.

### Article Analysis and Coding

Articles were initially grouped into either cross-sectional and longitudinal observational studies or intervention studies corresponding to our two goals: (1) to investigate the relationships between social engagement and health factors (cognitive, emotional, physical); and (2) to review the evidence base for interventions targeting social engagement for PwMCI. For studies identified as relevant to our first goal, we listed out all of the measures used in each study. We then coded measures as either “social engagement,” “health,” or “other.” Each social engagement measure was further coded to correspond to the factors in our multidimensional framework, and each health measure was coded as cognitive, emotional, or physical. Although our review focused on health factors, it is worth noting that many studies used contextual factors as covariates in their analyses, such as age, sex, location, etc., and often fell into the “other “category. Intervention studies were identified and categorized based on if the intervention approach was in-person or technology-based.

## Results

We address our first goal by presenting findings from cross-sectional and longitudinal observational studies, within each of the three health factors. We then address our second goal by presenting findings from studies that have addressed social engagement as an intervention approach.

### Relationship Between Social Engagement and Health

#### Cognitive Health

Our search yielded 13 studies that examined the relationship between social engagement and cognitive health in PwMCI (50–62). Overall, these studies addressed elements related to both activity and network, with a fairly even distribution between the two.

##### Activity

Seven studies examined components related to social activity, namely type (50, 53, 55, 61, 62), frequency/duration (50, 51, 61, 62), and format (57). No studies included analyses related to purpose or content/topic.

Longitudinal studies that examined cognitive status for PwMCI at baseline observed that higher levels of social activity were associated with a lower risk of progression from mild to severe cognitive impairment (61) and a higher likelihood of reversion from MCI to normal cognition (55). Hughes and colleagues (61) specifically looked at composite measures of both frequency of social activity and types of activities. The authors found that a lower risk of cognitive decline was associated with greater frequency of social activity engagement at baseline, and a slower decline was associated with participation in a variety of social activities across time. A further examination of activity type indicated that participants who progressed were less likely to attend church or worship, less likely to work, and less likely to engage in social organization events (i.e., they participated in fewer formal activities). Shimada et al. (55) similarly found that the type of activity may specifically contribute to the positive effects of social engagement. They examined the rate of reversion from MCI to normal cognition over a 4-year period and found that individuals who did not revert to normal were engaged in social activities less frequently than those who reverted, specifically those who took cultural classes, participated in hobbies or sports activities, or attended meetings in the community. One study, however, did not find an association between the likelihood of conversion from MCI to dementia over a 7-year period and the number of hours spent on social activities (50).

Four cross-sectional studies examined group differences in social activity engagement between PwMCI and cognitively normal controls (51, 53, 57, 62). Nygård and Kottorp (53) found that PwMCI participated less frequently in social activities outside the home relative to cognitively normal controls. Deng and colleagues (51) examined how PwMCI differed from cognitively normal adults on social activities in mid- and late-life and found that those with MCI had significantly less participation in late-life social activities compared to those with normal cognition, but there was no group difference in mid-life social activity. Kotwal et al. (62) examined social engagement in persons with and without MCI and found that those with MCI engaged less frequently in community activities (attending religious services, attending group meetings, and volunteering), but did not differ from controls in frequency of socialization with friends and family. Zhaoyang et al. (57) was the only study to examine format of social activities, wherein participants were asked to provide ecological momentary assessments (EMAs) by responding to prompts on a smartphone five times a day for 2 weeks regarding their daily social interactions. For one of the prompts, participants provided all activities they had participated in over the past 3–4 h, and the frequency of in-person vs. online social activities were summed. PwMCI had 31% lower odds of having in-person socializing each day than those without, but there was not a significant difference in online socializing activities.

Together these studies suggest that PwMCI tended to participate less frequently in social activities, particularly when those activities occurred outside the home, even when controlling for other factors (e.g., sex, race, education). Longitudinal studies indicated that increased frequency of participation in a variety of social activities, especially formal activities with some level of community involvement, may help preserve or even improve cognition and slow progression or reduce risk of dementia in those who already have a diagnosis of MCI.

##### Network

Nine studies examined components of social network, including frequency of interactions (55, 57, 59), closeness (52, 56, 57), size of network (56, 57, 59, 60, 62), and quality of interactions (54, 57–59, 62).

A longitudinal study that evaluated network size based on living situation and marital status (60) showed that living alone was significantly associated with greater risk of developing dementia, whereas being married/living with a spouse or living with a relative or caregiver was associated with lower risk of developing dementia. Another longitudinal study by Zhang et al. (56) found that having a larger social network was significantly associated with a decreased risk of conversion from MCI to probable dementia. Two cross-sectional studies of network size showed that PwMCI had smaller network sizes compared to cognitively normal individuals (59, 62). The study by Fankhauser et al. (59), which grouped PwMCI and Alzheimer's disease (AD) dementia together, found that network size (i.e., number of social contacts) was positively correlated with cognitive function, measured using the Mini-Mental State Examination. Kotwal et al. (62) examined the proportion of close relationships, described by the authors as network density, in addition to network size and found that lower cognition was associated with greater network density for MCI, with a higher proportion of familial relationships. The authors propose that this could reflect that (i) cognitive challenges make it more difficult to maintain varied social ties (and thus results in a smaller, denser network), or (ii) a dense, family-focused network serves as a compensatory mechanism where this network can help to monitor and support the cognitively impaired individual. These findings suggest the importance of evaluating not just network size but also closeness in the MCI population. Zhaoyang et al. (57) measured social network size and did not find a difference between cognitively normal older adults and those with MCI; however, this only included the number of close relationships (spouse, family members, and friends).

Zhaoyang et al. (57) examined closeness of social relationships using both EMAs and retrospective global measures that they developed. The EMAs showed that those with MCI had 30% lower odds of interacting with acquaintances, but did not show a difference with other closer social ties (family, friends etc.). Additionally, their global measure of social network included four questions about the composition of their social relationships, and no difference was found between MCI and non-MCI groups. Zhang et al. (56) examined closeness and roles of social ties but did not find a relationship between closeness of ties and risk of converting from MCI to dementia. One qualitative study addressed closeness through their efforts to identify perceived social determinants of health among PwMCI and their care partners (52). Thematic analysis of the dyads' semi-structured interviews revealed a theme of closeness as “connecting with neighbors and community.” This was characterized by camaraderie and helpfulness of neighbors, feelings of connectedness with others in the community, and the recurrent/weekly roles of community, family, and church events for PwMCI and their care partners. The authors discussed the possibility that such interactions promote feelings of social connectedness and engagement, which may help to promote cognitive health.

With regard to the frequency of social contacts, Fankhauser and colleagues (59) asked participants to provide the number of children, siblings, relatives, and friends or acquaintances they have contact with, and the frequency of contact. Frequency of social contacts did not differ between persons with cognitive impairment (both MCI and AD) and cognitively normal controls. In contrast, Zhaoyang et al. (57) found that PwMCI had 11% lower odds of participating in social interactions than those without MCI when measured by EMAs. However, no difference in frequency of social interactions was observed when measured with a retrospective global measure. Shimada et al. (55) used a single yes/no question to determine if the participants talked with people every day (format was not specified) and found that PwMCI who reverted to normal cognition engaged in more daily conversations than those who did not revert.

Studies on quality of interactions have examined satisfaction with network, social support, social strain, and negative vs. positive social interactions. Fankhauser et al. (59) did not find a difference in satisfaction with social contact between persons with cognitive impairment (a combination of MCI and AD) and cognitively normal controls. However, they did observe lower levels of social support in persons with cognitive impairment compared to controls. dos Santos et al. (58) similarly found that PwMCI scored worse than controls on a multidimensional scale of social support. Kotwal et al. (62) also observed an association between cognitive status and social support; however, this was moderated by sex, whereby women with lower cognitive scores perceived less social support, but there was no difference for men. They also found that participants with lower cognitive scores perceived less social strain than cognitively normal individuals, perhaps due to reduced social demand from those in their network. Zhaoyang et al. (57) observed that PwMCI had lower odds (14%) of having positive social interactions each day as measured by EMAs, and that the MCI group scored significantly higher on a measure of social strain than the non-MCI group, but scored the same on a measure of social support. Finally, a qualitative study by Renn et al. (54) asked PwMCI to take photographs over the course of 1 week that reflected important aspects of their day-to-day life and then conducted semi-structured interviews using the photographs. Following thematic analysis of these interviews, the importance of social support was a common theme, with participants emphasizing the need for familial support as well as support from and engagement with friends.

In summary, compared to cognitively healthy older adults, PwMCI may have smaller social networks composed of closer relationships and may not feel as socially supported compared to those experiencing typical cognitive aging.

#### Emotional Health

Four studies examined the relationship between social engagement and emotional health in MCI (63–66). Of these, only one study examined social activity (63), with the other three focusing on social network.

##### Activity

Amano et al. (63) identified patterns of social engagement by type (informal and formal) and found no relationship between presence of depressive symptoms and activity type.

##### Network

Three cross-sectional studies examined associations between social networks and emotional health in MCI. Kang and Lee (64) found that higher levels of social support were correlated with reduced depressive symptoms. Additionally, when including various health factors (somatic symptoms, sleep, functional ability), only social support and depression were significant predictors of overall quality of life. Another study found that social network size was correlated with overall quality of life in both persons with and without MCI, but the association was stronger in the MCI group (65). Yates et al. (66) examined how social networks might mediate the relationship between mood (including both anxiety and depression) and the presence of MCI. They noted that PwMCI had greater odds of having anxiety or depression, and generally had lower social network scores on the Lubben Social Network Scale (67), which probes number and frequency of social contacts within two different degrees of closeness (family and friends). They also found that social network score mediated the relationship between mood and MCI, with full mediation achieved only when both the family and friends subscales were included, indicating the importance of both types of relationships for PwMCI.

Overall, there was a relationship between emotional health and both the structure (e.g., size) and function (e.g., quality) of a person's network. Larger networks and more frequent, high quality interactions may be important for overall mood and quality of life in PwMCI.

#### Physical Health

Our search yielded six studies that examined the relationship between social engagement and physical health (63, 64, 68–71). The majority of these studies examined the relationship between physical health and social activity, with only one study examining social network (64).

##### Activity

Five studies addressed physical health as related to social activity. Four of these studies examined activity frequency (68–71) and one examined activity type (63).

Two studies by the same group using the same cohort of participants examined frequency of participation across nine different social activities and its relation to mobility (69) and fall rate (70) in cognitively normal older adults and PwMCI. Although their measure covered a range of different formal and informal social activity types (e.g., go out with others in public places, invite others to your home, provide care to others, volunteer), they did not analyze the effects of type of activity on mobility. In general, they found that MCI was associated with reduced mobility (quantified using both an objective and subjective measure), and activity frequency mediated this relationship (69). Similarly, MCI was strongly associated with number of falls (after adjusting for covariates), and activity frequency (dichotomized into low vs. high) moderated this relationship (70). For PwMCI, low activity frequency was associated with higher fall rate; however, if they had high levels of activity frequency, the association was no longer present. Correspondingly, a study by Gorenko and colleagues (68) found that social activity frequency moderated the association between gait velocity and cognitive status (MCI vs. healthy), whereas physical engagement did not have an effect on this relationship. For those with lower social activity frequency scores, gait velocity significantly predicted cognitive status, whereas for those with higher social activity frequency scores, this relationship was not present. The authors suggest that one potential mechanism underlying the relationship between social engagement, physical health, and cognition is the shared link with inflammation and dysregulation of a stress response. There is also emerging longitudinal evidence that social activity may mediate the link between health conditions (e.g., peptic ulcer recurrence) and sleep quality in PwMCI (71).

A study by Amano and colleagues (63) found that for PwMCI, health factors including self-rated health, number of chronic conditions, and activities of daily living, were significantly associated with type of social engagement (formal vs. informal). Those with higher self-rated health were more likely to engage in formal and informal types of social engagement.

Overall, engaging in social activities had a positive effect on physical health for PwMCI. Specifically, frequency of social activity participation, and participating in both formal and informal activity types, may be related to better physical health in PwMCI.

##### Network

Only one of the studies from our search related physical health to factors of social network in MCI. Kang and Lee (64) examined the association between social support (i.e., higher quality social network ties), somatic symptoms, and sleep quality within a group of PwMCI. They found that those with higher levels of social support had reduced somatic symptoms and better sleep quality, suggesting a relationship between the quality of a person's network and physical functioning.

### Interventions

Three interventions targeted social engagement in PwMCI (72–74). One used an in-person approach (74), whereas the other two used technology-mediated approaches.

#### In-person

The randomized controlled trial (RCT) conducted by Rovner et al. (74) was designed to prevent cognitive and functional decline among Black PwMCI. Participants in the intervention group attended five in-home behavioral activation therapy sessions (60 min each) over 4 months, followed by six maintenance sessions over the next 20 months. The therapy sessions consisted of goal setting and action plans to increase engagement in cognitive, physical, and social activities. They compared this approach to an active control group receiving standard supportive therapy. The primary outcome measure was cognitive functioning, measured with a single verbal list learning test, with a secondary outcome of physical health status, measured by functional decline. Although increasing social activity was part of the goal setting and action plans, there was no outcome measure related specifically to social activity. In general, they found that the intervention group maintained cognitive and physical functioning, whereas the active control group showed cognitive and functional decline.

#### Technology-Based

One study examined the feasibility of a virtual pet companion in increasing health outcomes for PwMCI (72). Ten female participants were given a tablet with a virtual pet, such as a dog, displayed on the screen. The device was connected to a call center with trained staff who would listen to the participant and type out responses that were read aloud by the virtual pet. Participants used the companion and reported that they appreciated its presence. Participants scored higher on measures of global cognition and social support and reported reduced depressive symptoms after having the virtual pet for 3 months. However, this study did not have a control group, and had a small sample size.

Another study used a technology-based platform to implement a multimodal RCT for PwMCI, where increasing social engagement was one of the intervention approaches (73). Participants in the intervention group had daily 30-min face-to-face communications using a web-enabled conversational system, whereas those in the control group received weekly telephone calls during which they were asked what social engagement activities they engaged in that week. The primary outcome was change in cognitive function, with a secondary outcome measure of loneliness. Both the intervention and control groups included persons with and without MCI. Following the intervention, those without MCI showed improvement in verbal fluency scores, whereas PwMCI did not have any significant effects. There was, however, a trend toward increased psychomotor speed for PwMCI. There was no difference between the intervention and control groups on a three-item loneliness scale.

Overall, our search identified few studies in relation to targeting social engagement in MCI. The approaches and targets varied, making it difficult to compare outcomes across studies. However, these results provide emerging evidence to support the benefit of social engagement for PwMCI.

## Discussion

### Summary of Findings

Our narrative review of the literature largely suggests that there are associations between social engagement and health factors in MCI, but very few intervention studies have targeted social engagement in this population. Positive associations were found across all three health factors (cognitive, emotional, physical) and social engagement, with higher levels of social engagement associated with better health, either directly or through mediating/moderating relationships. However, it is important to note that many of the relationships reported in the literature are derived from cross-sectional data, making it difficult to ascertain the direction of the effects.

The majority of the studies examined the relationship between social engagement and cognition, which is likely because the primary concern of PwMCI is declining cognition. However, the handful of studies examining physical and emotional components indicate that social engagement plays a role in supporting these aspects of health for PwMCI as well and warrant further investigation.

Although the literature points to the importance of social engagement in MCI, very few interventions have targeted social engagement in this population. Social engagement was the primary focus of only one of the three intervention studies reviewed (72). Interestingly, despite the other two intervention studies including social engagement as part of their multipronged approach, the outcomes only focused on cognitive and physical functioning as opposed to social engagement. More work is needed to clarify the role of social engagement in PwMCI and to determine the most effective approaches for intervention.

### Gaps in Social Engagement Literature in Relation to Framework

#### Relationship Between Social Engagement and Health

**Figure 3:**
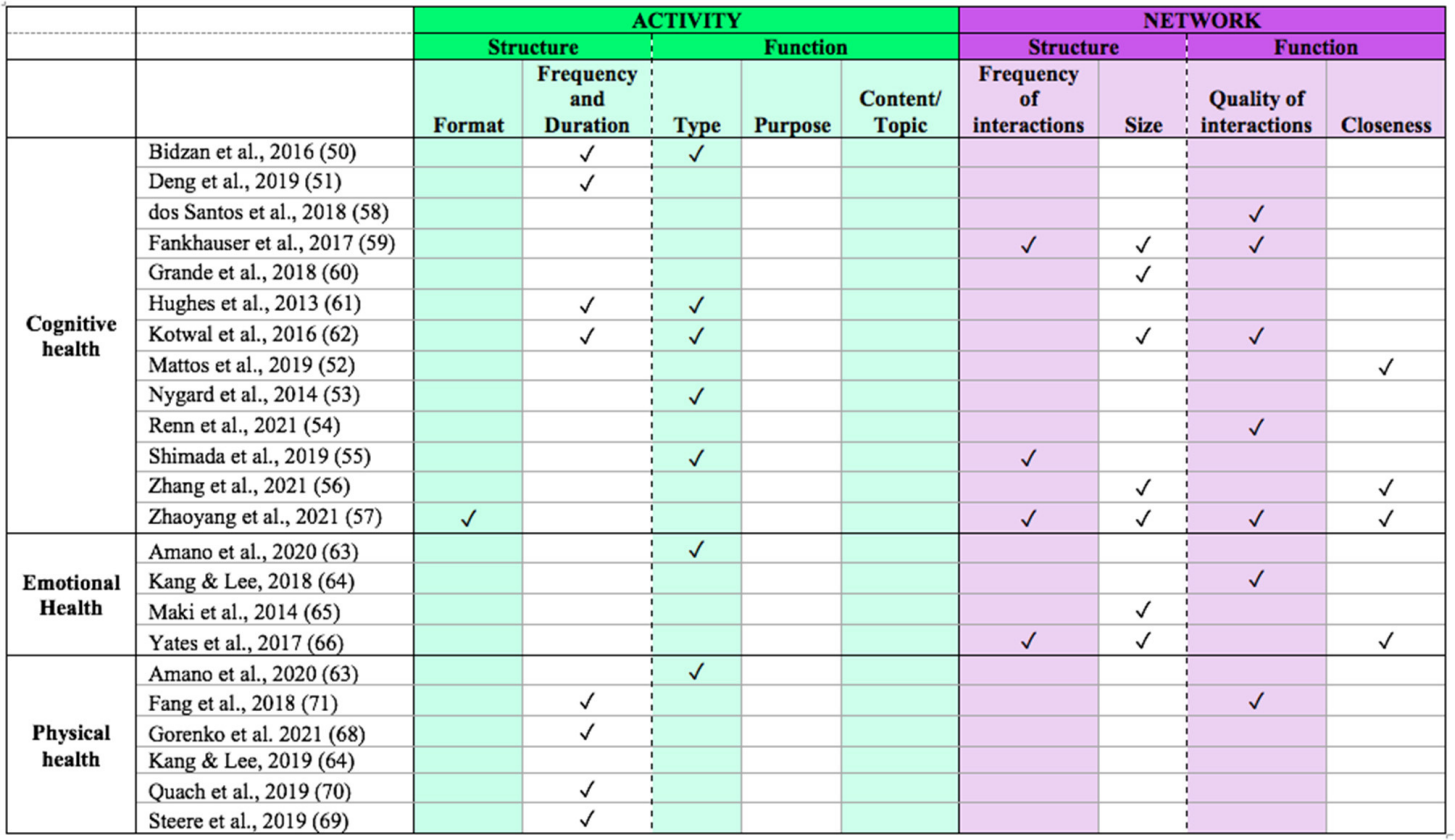
Framework components addressed by each study.

Keeping in mind that our narrative review provides a broad overview of research on social engagement in PwMCI, we have identified gaps in the current research in relation to our framework (see Figure 3). With regard to the two overarching dimensions of activity and network, we noted different patterns across the three health domains. For cognitive health, there was a fairly even distribution of studies examining social activity vs. social network. However, for emotional health the studies focused more on social network, whereas those on physical health focused more on social activity. Perhaps emotional health is thought to be linked to the ability to build relationships, whereas physical health is thought to play a more important role in activity participation. Nevertheless, characterizing both dimensions of social engagement across emotional and physical functioning would provide a more comprehensive understanding of the relationship between social engagement and health.

In further examining the components of activity and network across all health domains, it is clear that certain subcomponents have yet to be addressed in the context of MCI. With regard to activity, studies have primarily addressed frequency/duration and type. Although these components provide some insight into one aspect of their structure (frequency of engagement) and function (type of engagement such as formal or informal), further examination of the other components (e.g., content/topic, purpose) would provide additional insights about the effects of social engagement on health in these individuals. For example, it would be useful to understand if activities that serve different purposes, with various topics, would differentially affect health outcomes. Addressing these components directly would add valuable information. Taken together, such insights could inform the development of interventions, with targets that have the greatest impact on health and quality of life.

Evaluation or manipulation of the format of social activities (i.e., in-person vs. remote) is almost completely lacking in the literature. Given the increased adoption of more remote alternatives to activity participation, it will be important to directly measure differences between in-person vs. technology-based methods of engagement as they may affect health factors differently. For instance, physical health may be a limiting factor for participation in in-person activities, as these often require leaving the home, but this would not be a limiting factor if the activity was occurring remotely. Furthermore, the various components of social engagement may impact health differently depending on the format. For example, formal activities and community involvement may be important for maintaining cognitive health; however, whether such activities need to occur in-person or could be supported online has not been explored.

All four components related to network (frequency, size, quality, closeness) were addressed in at least one of the included studies. Size and quality of engagement were most frequently addressed. Size provides some information about the quantity of relationships, but it only captures part of the picture. Understanding how frequently a person actually interacts with those contacts would be critical to understand its relevance to social isolation and loneliness. This is especially true given that PwMCI appear to have shrinking social networks but may have closer and more frequent contact with network members (62).

Additionally, many studies did not examine more than one component–if they did, they both tended to fall within the same dimension (i.e., activity or network). In fact, only four studies measured components within both activity and network (55, 57, 62, 71). Given that social engagement includes both of these dimensions, it is crucial that studies measure at least one component from each to gain a more comprehensive understanding of social engagement in MCI. For example, our review suggests that PwMCI have reduced levels of social support, which may be important for supporting both physical and emotional health. However, we do not know how social activity participation may relate to this finding. Indeed, a recent review noted that a more comprehensive assessment of social engagement can be achieved by viewing it as multifactorial and assessing multiple components and their potential combined effects within a study, as opposed to examining a single component (37). It would be important for studies to characterize activities and networks across all the various structural and functional components to help tease apart the underlying mechanisms for how each relates to health, and to inform the development of interventions that can best serve the MCI population.

#### Interventions

The evidence about the effectiveness of social engagement interventions for PwMCI is extremely limited. Our search yielded three studies but only two addressed social engagement as an intervention method *and* assessed social health outcomes. One study measured changes in cognitive activity frequency but not social activity (74), and two measured changes in quality of network [loneliness (73) and social support (72)]. Future work should include a more comprehensive assessment of social engagement outcomes across the two dimensions (social activity and network). Also, all three of the intervention studies identified by our search manipulated structural components [increase social activity frequency (74); provide a new “relationship” (72); increase social activity and network size (73)]. The extent to which manipulation of functional components such as type, purpose, and content/topic impacts social engagement in PwMCI warrants further study.

With regard to technology, two of the three intervention studies used technology-mediated methods to deliver interventions, but the extent to which these technologies catered to the needs of older adults with cognitive impairment remains unclear. Some studies suggest there is acceptance of technology within the MCI population (75, 76), but optimizing technology for older adults with MCI before they are used for intervention needs careful consideration.

### Measurement Issues and Recommendations

Measures used to evaluate social engagement were highly varied across studies, making it difficult to compare findings. For example, unless the study was completed by the same group of authors, no two studies used the same measure of social activity frequency. Some utilized a fully developed assessment, such as Quach et al. (70) who utilized a subsection of the Late Life Function and Disability Instrument, whereas others used a few questions developed in-house [e.g., (54, 61)]. This inconsistency may be due in part to the lack of standardized and validated measures of social activity. Social network measures tend to be more consistent, with validated measures such as the Lubben Social Network Index (67) and The Multidimensional Scale of Perceived Social Support (77) being commonly used measures that capture the structure and function of networks, respectively. Examining each of the factors that constitute social engagement is important to tease apart how each may impact or be influenced by health in MCI; however, a single multidimensional scale that fully captures social engagement would also be beneficial (37).

Additionally, it would be useful to utilize more diverse measures of social engagement. The most common form of measurement across studies was a global questionnaire with quantitative scales. However, as pointed out by Zhaoyang et al. (57), given that PwMCI have difficulties with accurate recall, comparisons of activities between persons with and without MCI that require recall from memory may lead to misleading findings unless corroborated by informants. Alternative methods, such as EMAs that collect responses about the present moment at frequent intervals without requiring a recall from memory (57), and semi-structured interviews that collect rich information about an individual's experiences (52, 54) corroborated by informant interviews, can be used to supplement data obtained from validated measures.

Lastly, a barrier to integrating findings from studies of PwMCI relates to the inherent heterogeneity within this population, and the inconsistencies across studies regarding how MCI is diagnosed or defined. For example, some studies relied primarily on a single cognitive screening tool (62–64). Even if a formal diagnosis is not possible, more than one measure of cognitive status should be used to define participants as having MCI and their characteristics should be clearly described.

In summary, we recommend a more comprehensive examination of social engagement that samples both the activity and network dimensions more fully. Future studies should evaluate how the content, purpose, and format of social activity in PwMCI is linked to cognitive, emotional, and physical health. Research characterizing the impact of the quality of interactions and closeness within the network dimension could advance understanding of the extent to which enrichment activities should be planned for PwMCI. Our understanding of the benefits of social engagement for PwMCI is fairly limited. Carefully designing interventions to address various components of social engagement and evaluating outcomes using a battery of measures would be important to establish the value of such interventions for promoting health outcomes. Our framework (Figure 1) elucidates the components to consider in the design of social engagement interventions. Finally, as technology access becomes more ubiquitous and affordable, developing technology-based social engagement interventions with broader reach could serve a significant role in addressing social isolation in PwMCI.

## Author Contributions

RM and WR conceived the primary research question for the study. EL, LN, and QN drafted the initial framework with guidance from RM and WR. EL and LN assessed the papers for inclusion and interpreted the results. EL, LN, and RM drafted the initial manuscript. WR and QN provided input and revision to the manuscript. All authors contributed to the article and approved the submitted version.

## Funding

This research was supported in part by the National Institutes of Health (National Institute on Aging) Grant R44 AG059450, entitled Enhancing Quality of Life for Older Adults with and without MCI through Social Engagement over Video Technology, as well as by a grant from the National Institute on Disability, Independent Living, and Rehabilitation Research (NIDILRR Grant Number 90REGE0012) under the auspices of the Rehabilitation and Engineering Research Center on Enhancing Neurocognitive Health, Abilities, Networks, and Community Engagement (ENHANCE; www.enhance-rerc.org).

## Conflict of Interest

LN was employed by the company iN2L. The remaining authors declare that the research was conducted in the absence of any commercial or financial relationships that could be construed as a potential conflict of interest.

## Publisher's Note

All claims expressed in this article are solely those of the authors and do not necessarily represent those of their affiliated organizations, or those of the publisher, the editors and the reviewers. Any product that may be evaluated in this article, or claim that may be made by its manufacturer, is not guaranteed or endorsed by the publisher.
